# Vertical Stiffness, Pelvic Tilt Range and Jump Height during Vertical Jumping in Youth Soccer: Influence of Age, Sex and the Playing Position

**DOI:** 10.5114/jhk/204868

**Published:** 2025-11-19

**Authors:** Rafael Martínez, Alejandro Sanz, Florentino Huertas, Paul J. Byrne, Javier Martínez-Gramage

**Affiliations:** 1 Doctoral School, Catholic University of Valencia “San Vicente Mártir”, Valencia, Spain.; 2 Faculty of Physical Education & Sport Sciences, Catholic University of Valencia “San Vicente Mártir”, Valencia, Spain.; 3 Department of Health and Sport Sciences, Faculty of Health Science, South East Technological University (Kilkenny Road Campus), Carlow, Ireland.; 4 Department of Physiotherapy, Faculty of Health Sciences, Universidad Cardenal Herrera-CEU, CEU Universities, Valencia, Spain.

**Keywords:** age, jumping performance, stiffness, sex, pelvic tilt

## Abstract

This study aimed to examine the influence of sex, age, and the playing position on vertical stiffness, pelvic tilt range, and jump height during vertical jumping in young soccer players. A total of 542 players (129 females, 413 males) aged 12 to 19 years from elite soccer academies participated. Players were divided into U14, U16, and U19 categories, and jump height, vertical stiffness, and pelvic tilt range were evaluated using the countermovement jump (CMJ) with an inertial sensor. Results revealed that vertical stiffness and jump height were significantly higher in males than females (p < 0.001) and significantly increased with age in males only from U14 to U16, U16 to U19 and U14 to U19 (p < 0.01 to p = 0.015). Pelvic tilt range was significantly greater in females only (p < 0.001), with forwards showing larger pelvic tilt range than midfielders (p = 0.023), though no differences were found by age. The analysis of the playing position showed no significant differences in vertical stiffness or jump height, suggesting that these variables were more influenced by biological and sex-related factors rather than positional roles during developmental stages. These findings provide normative data that can aid in the design of individualized training programs aimed at optimizing performance and reducing injury risk in youth soccer players.

## Introduction

Physical demands in soccer vary depending on the performance level, the player’s position, and the age category (Deprez et al., 2015). The study of conditional factors (e.g., strength, speed, and agility) affecting elite soccer performance has become increasingly important, both in adult players (Maly et al., 2018) and in youth athletes (Dragijsky et al., 2017). Therefore, the objectives, content, and training methodologies aimed at improving physical condition should be adapted to the athlete’s developmental stage. This is especially relevant in soccer academies, where optimizing talent identification and development processes has become increasingly important, with substantial financial, human, and material resources invested to maximize performance and minimize injury risks (Ginés et al., 2023; Sanz et al., 2020).

Among the most frequent actions in soccer there are jumps, sprints, and changes of direction, which are critical actions preceding goal-scoring opportunities (Barrera-Domínguez et al., 2025; Gouveia et al., 2023). A key musculoskeletal property for optimizing efficiency in these actions is stiffness (Brazier et al., 2014), which is defined as the deformation experienced by a structure in response to an applied force. A previous study (Maloney and Fletcher, 2021) has differentiated among three types of stiffness: vertical stiffness (K_vert_), defined as the vertical displacement of the center of mass in response to the vertical ground reaction force during an action in the sagittal plane; leg stiffness (K_leg_), which refers to the compression of the leg spring in response to force in any plane or direction; and joint stiffness (K_joint_), which corresponds to the angular displacement of a joint in response to the moment of force at the joint.

From a practical standpoint, vertical stiffness (K_vert_) is the most relevant capacity in sports like soccer, where vertical jumping is highly significant for performance (Brazier et al., 2014). A previous study has shown that K_vert_ provides valuable insights into a soccer player’s stiffness profile, with strong associations to performance in actions such as jumping, sprinting or changing the direction (Maloney and Fletcher, 2021). Practically speaking, optimal performance in running or jumping requires an appropriate level of K_vert_ in the lower limbs, enabling the absorption of ground reaction forces (GRFs) and the storage and reuse of elastic energy. Previous research (Pruyn et al., 2014) has reported that higher K_vert_ values are linked to improved sprint performance, greater acceleration, and higher jumps in activities involving rapid stretch-shortening cycles. However, another study has found that excessively high K_vert_ values may increase the risk of bone injuries such as stress fractures and osteoarthritis (Butler et al., 2003). Conversely, deficits in K_vert_ may lead to excessive joint range of motion, potentially causing soft tissue injuries (Granata et al., 2002). Another study has reported that high hamstring stiffness can act as a protective factor against anterior cruciate ligament (ACL) injuries in both physically active men and women (Blackburn et al., 2013). However, none of the aforementioned studies have provided specific evidence regarding optimal, deficient or excessive K_vert_ ranges in various contexts. Despite the critical role of physical and biomechanical variables in youth soccer performance, there is a notable lack of research examining vertical stiffness, pelvic tilt range, and jump height during key developmental stages. Addressing this gap is essential for designing targeted training programs and injury prevention strategies tailored to the specific needs of youth soccer players at different stages of development. Filling this gap with a large sample size, as per the present study, is particularly important for establishing reliable reference values.

Another key factor influencing performance and predicting injury rates in soccer players is pelvic dynamics in the sagittal plane (Mendiguchia et al., 2024). The pelvis plays a stabilizing role and transfers energy between the lower limb and the rest of the body. Women generally exhibit a greater anterior pelvic tilt than men (Kernozek et al., 2005) and are between two and eight times more likely to suffer ACL injuries (Márquez et al., 2017). There is evidence supporting the association between the pelvic tilt and ACL injury risk (Hertel et al., 2004), as well as hamstring injuries in soccer players (Bramah et al., 2023). During sprinting, increased pelvic anteversion and trunk flexion have been observed, accompanied by greater elongation at the biceps femoris myotendinous junction during both the late stance and late swing phases (Higashihara et al., 2015).

In youth athlete development stages, especially during the period near peak height velocity (PHV), adolescents experience their highest growth rate in height (Corso, 2018). During this stage, it is especially important to monitor training load volume and intensity, as they can affect harmonious and balanced growth, reducing the risk of future injuries and helping achieve performance goals for this stage (Ford et al., 2011).

To date and to the best of the authors' knowledge, there is **no literature** examining the evolution of vertical stiffness, pelvic tilt range, and their impact on performance across different developmental stages in soccer. Categories such as U14 for males and U12 for females are particularly sensitive, as they correspond to the PHV, during which there is a sudden increase in the growth rate of limbs and body mass (Philippaerts et al., 2006). Malina (2010) has previously concluded that these stages present a high risk of injury, which should be considered by coaches when planning strength, power, and coordination training.

Given the lack of studies on youth soccer players that consider important variables such as sex or the playing position and include vertical stiffness and pelvic tilt range, the present study aimed to provide novel insights into the normative values of these variables and their relationship with jumping ability in soccer players during the developmental stages (12–19 years old). Establishing these reference values would enable the identification of players exhibiting deficits in the aforementioned variables, thereby facilitating the optimization of training protocols to enhance performance and mitigate injury risk.

## Methods

### Participants

A total of 542 young soccer players (129 females and 413 males) from 35 teams belonging to four elite soccer academies in the Valencian Community (Spain) voluntarily participated in the study. Participants were divided and classified according to their age category (U14, U16, U19) (Table 1). All players followed a training regimen with a minimum of three sessions per week and one competitive match per week (Sanz et al., 2020). The medical staff from each club provided formal medical clearance for each player, confirming their fitness to participate in the study. Parents and players signed agreements for the use of their data for both internal and external research purposes of the club. Additionally, both parents and players signed informed consent and assent forms, respectively, with detailed players’ information. This research received approval from the Catholic University of Valencia “San Vicente Mártir” ethics committee, Valencia, Spain (approval code: UCV/2022-2023/092; approval date: 14 February 2023), in accordance with the ethical guidelines of the Declaration of Helsinki (2013).

**Table 1:** Descriptive results (mean ±SD) of dependent variables (vertical stiffness, range pelvic tilt and jump height) according to sex, the age category and the playing position.

Sex	Age category	Position	Age (years)	Body mass (kg)	Vertical Stiffness (N/m)	Pelvic Tilt Range (º) (º)	Jump height (cm)
FEMALE (n = 129)	U14 (n = 47)	GK (n = 6)	13.23 (± 0.809)	49.20 (± 6.6))	1267.57 (± 319.35)	61.5 (± 7.6)	24.33 (± 1.75)
		DEF (n = 18)	13.40 (± 0.62)	49.47 (± 7.13)	1262.53 (± 374.02)	59.72 (± 10.73)	23.16 (± 2.77)
		MID (n = 10)	13.35 (± 0.57)	49.37 (± 5.06)	1528.51 (± 478.22)	51.9 (± 8.6)	23.5 (± 3.13)
		FOR (n = 13)	13.23 (± 0.60)	45.82 (± 7.62)	1168.21 (± 461.51)	64.76 (± 11.62)#	23.84 (± 4.67)
	U16 (n = 39)	GK (n = 3)	15.59 (± 0.47)	60.73 (± 7.12)	1338.81 (± 516.27)	60.0 (± 9.8)	24.33 (± 1.15)
		DEF (n = 13)	15.28 (± 0.65)	55.03 (± 3.66)	1503.53 (± 678.90)	63.53 (± 8.45)	21.07 (± 4.19)
		MID (n = 12)	15.36 (± 0.49)	52.60 (± 3.78)	1295.59 (± 210.77)	60.41 (± 8.33)	22.75 (± 3.44)
		FOR (n = 11)	15.32 (± 0.46)	54.98 (± 5.06)	1191.38 (± 278.66)	64.63 (± 8.55)#	24.09 (± 2.73)
	U19 (n = 43)	GK (n = 4)	17.45 (± 1.18)	60.80 (± 7.23)	1518.80 (± 229.67)	54.5 (± 6.35)	25.75 (± 2.21)
		DEF (n = 12)	17.96 (± 0.98)	57.89 (± 5.36)	1551.07 (± 346.63)	58.91 (± 9.65)	24.91 (± 4.68)
		MID (n = 15)	17.83 (± 0.79)	59.13 (± 5.01)	1563.01 (± 333.73)	60.46 (± 5.44)	23.53 (± 3.11)
		FOR (n = 12)	17.99 (± 0.91)	57.52(± 6.22)	1344.09 (± 460.24)	63.75 (± 10.58)#	24.0 (± 2.69)
MALE (n = 413)	U14 (n = 139)	GK (n = 12)	13.64 (± 0.56)	61.37 (± 12.65)	1860.73 (± 983.84)	52.33 (± 14.25)	26.33 (± 5.97)
		DEF (n = 49)	13.51 (± 0.57)	52.33 (± 9.11)	1346.68 (± 392.06)	56.71 (± 11.02)	26.30 (± 4.30)
		MID (n = 41)	13.53 (± 0.57)	50.60 (± 9.60)	1346.70 (± 364.44)	53.22 (± 8.31)	25.75 (± 4.28)
		FOR (n = 37)	13.29 (± 0.51)	48.73 (± 8.92)	1379.27 (± 556.24)	55.91 (± 10.28)	26.48 (± 3.85)
	U16 (n = 123)	GK (n = 12)	15.52 (± 0.56)	69.73 (± 6.52)	1357.97 (± 325.91)	54.75 (± 10.25)	30.41 (± 4.27)
		DEF (n = 41)	15.46 (± 0.53)	63.77 (± 7.36)	1552.22 (± 506.98)	55.12 (± 8.90)	30.26 (± 4.95)
		MID (n = 28)	15.56 (0.46)	63.17 (± 5.94)	1655.89 (± 346.60)	54.21 (± 7.80)	30.89 (± 5.19)
		FOR (n = 42)	15.37 (± 0.55)	62.35 (± 6.79)	1701.90 (± 673.83)	56.11 (± 9.43)	29.07 (± 5.26)
	U19 (n = 151)	GK (n = 16)	17.68 (± 0.71)	74.48 (± 9.32)	1615.05 (± 417.79)	59.37 (± 10.07)	33.5 (± 5.39)
		DEF (n = 51)	17.70 (± 0.82)	72.13 (± 5.42)	1743.62 (± 406.43)	55.23 (± 6.38)	31.64 (± 3.78)
		MID (n = 39)	17.89 (± 0.92)	69.63 (± 4.87)	1736.02 (± 478.06)	54.56 (± 8.19)	29.64 (± 4.06)
		FOR (n = 45)	17.80 (± 0.79)	70.46 (± 6.68)	1869.72(± 484.43)	52.86 (± 7.99)	32.02 (± 5.06)

# p < 0.001: statistically significant differences by playing position, only between midfielders and forwards in females

### Design and Procedures

#### Data Collection

After obtaining approval from the respective club representatives, informational meetings were held with coaches to explain the study’s procedures. Before conducting the assessments, team representatives recorded the following data for each player: body mass (kg), leg dominance (the leg chosen to kick a penalty), and the predominant specific field position. Birthdates were also recorded, and players were grouped according to their age category following the criteria established by the Royal Spanish Soccer Federation.

#### Procedures

To minimize the potential impact of fatigue on the measurements, all participants and their coaches were instructed to refrain from intense physical activity or training sessions during the 24 hours preceding the testing sessions. This protocol aimed to ensure that players were well-rested and in optimal physical condition for reliable and valid data collection (Muyor and Arrabal-Campos, 2016). Players were scheduled to arrive 30 min before the start of the training session. The measurements were collected under standardized conditions (18 ± 2°C) on a hard indoor surface at each club’s sports facility. The measurements were carried out from March to April, during the competitive season.

All the dependent variables were assessed using the countermovement jump (CMJ). Participants were asked to perform barefoot and were instructed to jump as quickly as possible to reach maximum height in each trial. Participants maintained a standing position at the start of the jump and, before jumping, performed a countermovement until the knees were flexed approximately to 90°. Three CMJs were performed with bipodal support, with 30-s rest intervals between each jump. During the CMJs, hands were placed on the pelvis to eliminate the arm swing. In addition to jump height, two additional dependent variables were evaluated: vertical stiffness and pelvic tilt range. The trial with the highest jump height was used for analysis. Criteria for CMJ rest intervals and jump protocol procedures followed previous recommendation (Parra et al., 2024).

Jump height (cm) was defined as the maximum height reached, measured from the tip of the foot to the ground, being calculated using equation 1, where FT is the flight time in seconds (Lloyd et al., 2015).

(FT2 1.226) x 100 (1)

Vertical stiffness (N/m), was defined as the resistance to vertical displacement after utilizing ground reaction forces, and it was calculated using equation 2, where M is the participant's mass in kilograms, FT refers to the flight time in seconds, and CT is the contact time in seconds (Dalleau et al., 2004).


(2) $$\frac{M\times \pi \left(FT+CT\right)}{contactTime^{2}\left(\frac{FT+CT}{\pi }-\frac{CT}{4}\right)}$$

Pelvic tilt range (º) was defined as the angular displacement of the pelvis in the sagittal plane throughout the entire jump execution.

**Figure 1:**
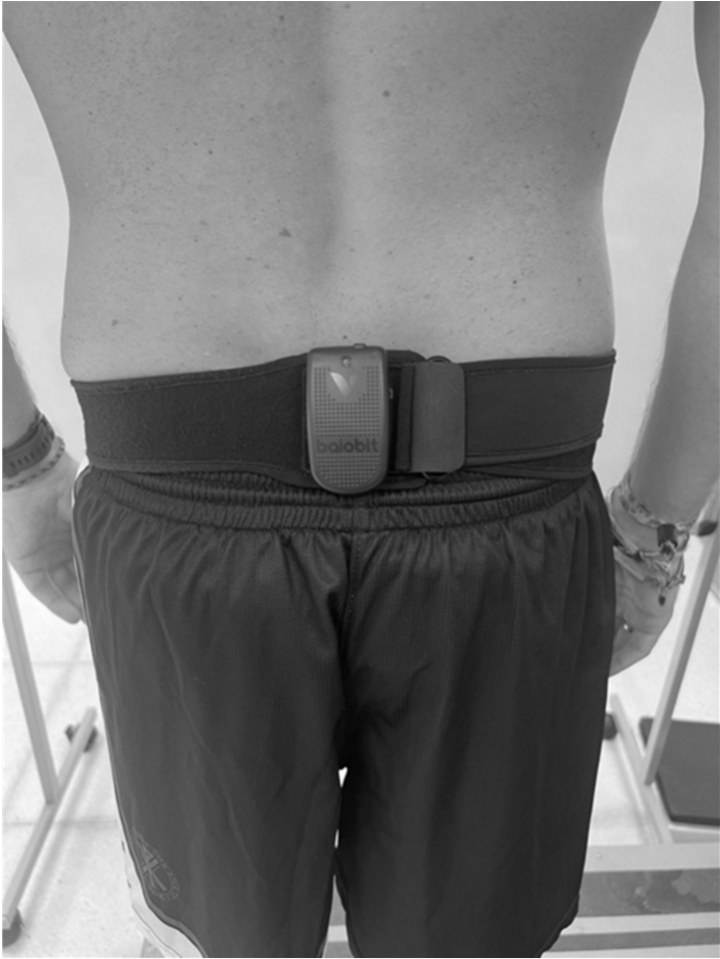
Placement of the IMU in participants S1.

The measuring instrument was a Baiobit sensor (Rivelo Srl—BTS Bioengineering Group, Milan, Italy), fitted with an ergonomic belt placed at the height of S1 (Figure 1). The Baiobit includes a triaxial accelerometer, a 13-bit triaxial magnetometer, and a triaxial gyroscope. All measurements were recorded at a sampling frequency of 200 Hz. A previous study has confirmed the Baiobit sensor's inter-instrument correlation coefficient ranging between 0.90 and 0.99, and an intra-instrument coefficient of variation of ≤ 2.5% (Camuncoli et al., 2022). The calibration was carried out according to the manufacturer's instructions. The sensor has been validated as a reliable tool for measuring the study variables (i.e., vertical stiffness, pelvic tilt, and jump height) when compared to the gold standard of force plates (Camuncoli et al., 2022). Its primary advantage lies in its accessibility and affordability, making it feasible for use in a wide range of clubs that may lack the resources to invest in a more expensive force plate system.

#### Statistical Analysis

The criteria for the exclusion of participants in the present study were as per Ishøi (2016). Seventeen players were excluded due to discomfort or muscular overload on the day of the evaluation and further 31 players were excluded due to a long-term lower-limb injury (≥ 6 weeks) in the two months preceding the measurements. This information was systematically gathered by the medical staff of each participating club. Additionally, nine players were excluded due to errors in the data collection device. Descriptive results were reported as mean values ± SD. With the remaining 485 players, a sensitivity analysis was conducted using G*power (Faul et al., 2007). The minimum effect size detectable for α = 0.05, and 1−β = 0.80 for four groups, was f = 0.14 (minimum detectable effects).

Differences in vertical stiffness, pelvic tilt range, and jump height based on independent variables (sex, age groups, and playing position) were evaluated using one-way analysis of variance (ANOVA). The partial squared effect size was also reported, with small (η^2^ > 0.01), moderate (η^2^ > 0.06), and strong (η^2^ > 0.14) effects (Field, 2009). Statistical significance was set at *p* ≤ 0.05. The statistical analysis was conducted using the JASP software (version 0.17.2, University of Amsterdam, Amsterdam, The Netherlands).

## Results

The mean values for vertical stiffness, pelvic tilt range, and maximum jump height by sex, age category, and playing position are presented in Table 1.

### Vertical Stiffness

**Figure 2:**
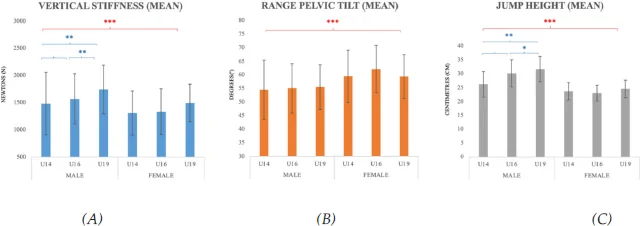
Means and SDs of vertical stiffness (A), range of the pelvic tilt (B) and maximum jump height (C) according to sex and the age category. (*) indicates significant differences (p < 0.05); (**) indicates significant differences (p < 0.01); (***) indicates significant differences (p < 0.001); the red color represents the differences by sex; the blue color represents the differences by age group

Our results showed significant differences by sex, with males exhibiting 16.1% higher stiffness values than females (*F*(1,540) = 19.19, *p* < 0.001, η^2^ = 0.034) (Figure 2A). Analyzing the sample as a whole, significant differences were found between age categories (*F*(1,540) = 21.82, *p* < 0.001, η^2^ = 0.075), with U19 athletes showing the highest vertical stiffness values, specifically 9.5% higher than U16 athletes (*p* = 0.004) and 19.5% higher than U14 athletes (*p* < 0.01). No significant differences were found based on the playing position (*p* = 0.83).

The following presents a detailed analysis by sex, age category, and playing position. In males, significant differences were found by age category (*F*(2,410) = 19.39, *p* < 0.001, η^2^ = 0.086), showing that stiffness increased with age (Figure 2A). U19 players displayed the highest vertical stiffness values, 8.93% higher than U16 players (*p* < 0.01) and 20.73% higher than U14 players (*p* < 0.01). U16 players had 12.9% higher values than U14 players (*p* < 0.01). No significant differences were found based on the playing position (*p* = 0.295).

In females, age did not significantly modify stiffness (*p* = 0.065), although stiffness increased with age in a similar pattern to that observed in males. Specifically, U19 players exhibited the highest vertical stiffness values, 10.42% higher than U16 players (*p* = 0.215) and 13.45% higher than U14 players (*p* = 0.063). U16 players showed stiffness values 3.36% higher than U14 players (*p* = 0.872). No significant differences were found in vertical stiffness by playing position (*p* = 0.103).

### Pelvic Tilt Range

As with stiffness, when analyzing the entire sample, significant differences in pelvic tilt range were found by sex, with females showing 9.5% higher values than males (*F*(1,540) = 38.91, *p* < 0.001, η^2^ = 0.067) (Figure 2B). In contrast, no differences were found by age category (*p* = 0.615) or playing position (*p* = 0.243).

When performing a detailed analysis by sex, age group, and playing position, no significant differences were found by age category in males (*p* = 0.923) or by playing position (*p* = 0.448).

Considering the total sample of female players, no significant differences were found by age category (*p* = 0.341). However, differences in pelvic tilt range were observed based on the playing position (*F*(3,125) = 2.995, *p* < 0.001, η^2^ = 0.067), with forwards showing values 9.5% higher than midfielders (*p* = 0.023).

### Jump Height

Analyzing the entire sample, our results showed significant differences by sex (*F*(1,540) = 139.63, *p* < 0.001, η^2^ = 0.205), with males achieving jump heights 19.52% greater than females (Figure 2C).

Regarding the detailed analysis by sex, significant differences were found in males for jump height based on the age category (*F*(2,410) = 48.79, *p* < 0.001, η^2^ = 0.192), with performance improving as age increased (Figure 2C). U19 males jumped 4.51% higher than U16 males (*p* = 0.015) and 16.67% higher than U14 males (*p* < 0.01). U16 males showed 12.72% higher performance than U14 males (*p* < 0.01). However, no significant differences were found by playing position in males (*p* = 0.202).

In females, no significant effects were found by age category (*p* = 0.123) or playing position (*p* = 0.338).

## Discussion

The present study provides new insights into the normative values of vertical stiffness, pelvic tilt range, and jump height in youth soccer players, differentiated by sex, age category, and playing position.

Our results regarding vertical stiffness show significant differences by both sex and age category, but not by playing position. Males exhibited higher vertical stiffness values compared to females, which is consistent with a previous study (Ward et al., 2019). However, it should be noted that in the aforementioned study, adult dancers and field sport athletes were the participants. Laffaye et al. (2014) suggested that these differences could be attributed to the superior neuromuscular capacity of males to store and reuse elastic energy during the stretch- shortening cycle, which directly impacts performance in dynamic maximal force actions (> 0.30 s) and reactive strength, which reflects the ability to quickly transition between eccentric and concentric muscle actions (Nuzzo et al., 2023). This greater capacity for generating eccentric force could indicate that males are more efficient at storing and using elastic energy during the pre-stretch phase of movement, a crucial mechanism for muscle stiffness (Laffaye et al., 2014).

Regarding the age category, there was a progressive increase in vertical stiffness with age, which was statistically significant in males starting from the U16 category. In females, although an increase in stiffness with age was observed, the differences were not statistically significant. The absence of significant stiffness changes in female players across U14–U19 categories may be attributed to smaller sample sizes, variability in biological maturation, and differences in training loads, potentially reducing statistical power. These results align with studies linking the increase in stiffness to PHV, where muscle properties are modified, leading to greater muscle stiffness (Charcharis et al., 2019).

As shown by Bloomfield et al. (2007), physical demands vary significantly between positions in elite adult soccer, suggesting the need for position-specific training programs. However, in the developmental stages observed in the present study, no significant differences in vertical stiffness were found by playing position. The discrepancy between findings in adult and youth soccer could be explained by the developmental approach in youth soccer, where early specialization in a specific position is avoided, and a more comprehensive development of physical and technical skills is encouraged.

Regarding pelvic tilt range, our results showed that this variable varied by sex but not by age. Consistently with a previous study on youth distance runners (Taylor-Haas et al., 2022), our female players exhibited greater pelvic tilt ranges than male players. Regarding age, no significant differences were found in either sex, suggesting that pelvic tilt range may be influenced by anatomical and biomechanical factors rather than physical development associated with age. However, it is noteworthy that female forwards showed a greater pelvic tilt range than female midfielders. The observed differences in the pelvic tilt, particularly among female players and female forwards, have significant implications for injury prevention. Female athletes are at higher risk of ACL injuries due to anatomical, physiological, and biomechanical factors such as increased joint laxity and neuromuscular control deficits. The anterior pelvic tilt has been linked to biomechanical changes, including increased knee valgus and altered hip and knee kinematics, which are associated with a heightened risk of ACL injuries (Di Paolo et al., 2023). Women tend to exhibit a greater range of hip internal rotation and pelvic tilt, further increasing their susceptibility to ACL injuries, particularly when engaging in dynamic knee valgus movements during play (Brophy et al., 2009).

Position-specific training programs tailored to these postural patterns could mitigate injury risk and enhance performance for female players (Oliva-Lozano et al., 2020). Core stability and hip mobility exercises should be incorporated into training routines to address these risk factors. Additionally, including pelvic tilt assessments in injury prevention programs could facilitate early identification of high-risk players, enabling the implementation of personalized strategies to reduce injury rates. When considering the influence of the anterior pelvic tilt on hamstring elongation, targeted strengthening exercises may contribute to reducing the risk of hamstring strains. To promote balanced muscle strengthening, these exercises (e.g., a lunge exercise for the proximal region and the Nordic hamstring exercise for the distal region) should address both the proximal and distal regions of the hamstrings (Mendiguchia et al., 2024).

Although the limited sample size of players in certain positions warrants caution in interpreting these findings, this pattern highlights the importance of considering the playing position when designing injury prevention programs, especially for female players, as certain positions may predispose them to a higher risk of injury related to pelvic biomechanics.

Finally, our results regarding motor performance, quantified as jump height, replicated previous findings regarding differences by sex (greater jump capacity in males) and age category (greater jump capacity in older players), but not by playing position (Lago-Peñas and Lago-Ballesteros, 2011). Positional differences are less pronounced in younger age groups, likely due to their ongoing musculoskeletal growth, and become more evident as players mature. This is primarily attributed to natural growth and maturation processes, which enhance muscle strength and neuromuscular coordination (Silva et al., 2022). Additionally, the absence of significant differences may be attributed to the limited time spent specializing in a specific position at these developmental stages, as players often rotate across multiple positions to foster overall skill development.

In terms of sex, a previous study has attributed these differences to the greater muscle mass and force generation capacity in males, which is associated with a better force-time relationship during jumping (Laffaye et al., 2014). Regarding the age category, we found that the U14 category in males showed the largest increase in jump capacity compared to U16 and U19. This could be explained by the increase in muscle mass and physical maturation that occurs during stages near PHV (Philippaerts et al., 2006). This finding suggests that training programs should consider these developmental differences to maximize jump capacity in young players.

From a practical application standpoint, the present study includes a large sample of youth soccer players in terms of males and females from U14 to U19 in relation to vertical jumping (Ma et al., 2025), where vertical stiffness and the pelvic tilt were recorded during this explosive movement. Furthermore, these age groups for both females and males have also been categorized according to playing positions, namely, goalkeepers, defenders, midfielders and forwards. These vertical jumping data are useful for coaches and sport science practitioners to have normative values to work from when designing training programs to improve physical characteristics and reduce injury risk in youth soccer players from U14 to U19 (Zabaloy et al., 2025).

For future research, it would be useful to explore how physical development and maturation affect these variables in larger samples and to analyze their evolution in adult soccer players, where positions are more stable. Further investigation into the relationship between pelvic tilt range and ACL injury risk, as well as the implications of positional rotation in formative stages, is also recommended.

The present study acknowledges the reliance on chronological age as a proxy for maturation, which may not fully capture the variability in physical and biomechanical characteristics during youth development. Biological maturation markers, such as PHV, could provide a more precise framework for interpreting the observed differences in vertical stiffness, pelvic tilt range, and jump height. Incorporating such markers in future studies would allow for a deeper understanding of the interplay between maturation and physical performance in youth soccer players. This approach could help refine training and injury prevention programs tailored to the developmental stage of each athlete. Also, the absence of significant differences in stiffness or jump height by playing position may be due to the limited positional specialization in youth players, who often rotate across roles to promote overall development. Additionally, differences in biological maturation between positions might have masked potential effects. Future studies focusing on adult players, where physical demands and position-specific characteristics are more defined, could provide a clearer understanding of these relationships.

## Conclusions

This study aimed to investigate vertical stiffness, pelvic tilt range, and jump height in youth soccer players, differentiated by sex, age, and their playing position. The findings revealed that vertical stiffness and jump height varied significantly by sex and age, with males and older age groups showing higher values. However, no significant differences were observed in vertical stiffness or jump height by playing position. Additionally, pelvic tilt range was significantly higher in females compared to males, but this variable did not show significant differences by age. However, it is important to interpret these data with caution due to the small sample size.

The results underscore the importance of considering both sex and age in designing training programs and injury prevention strategies for youth soccer players. The data provided here offer normative values that can help coaches tailor their approaches to enhance physical performance while reducing injury risks in youth players from U14 to U19.

Certain findings, such as the observed differences in pelvic tilt range, should be interpreted with caution due to potential limitations in sample sizes within subgroups and variations in training methodologies across the participating clubs. These factors may have influenced the consistency and comparability of the results.

Future research with larger, more balanced samples and standardized training protocols across clubs is recommended to reduce variability and provide more generalizable conclusions. We recommend longitudinal research to track PHV's effects on stiffness and the pelvic tilt across development and to evaluate positional differences in fully matured players, where roles are more defined. This will clarify how maturation and positional specialization modulate the relationship between pelvic tilt range and the risk of ACL injuries.
